# “The Dollar Store Got It Going On”: Understanding Food Shopping Patterns and Policy Preferences among Dollar Store Shoppers with Low Incomes

**DOI:** 10.1016/j.cdnut.2024.104457

**Published:** 2024-09-13

**Authors:** Alexandria E Reimold, Marissa G Hall, Shu Wen Ng, Lindsey Smith Taillie, Kurt M Ribisl, Emile L Charles, Shelley D Golden

**Affiliations:** 1 Department of Health Behavior, Gillings School of Global Public Health, University of North Carolina, Chapel Hill, NC, United States; 2 Carolina Population Center, University of North Carolina, Chapel Hill, NC, United States; 3 Lineberger Comprehensive Cancer Center, University of North Carolina, Chapel Hill, NC, United States; 4 Department of Nutrition, Gillings School of Global Public Health, University of North Carolina, Chapel Hill, NC, United States

**Keywords:** dollar stores, food purchases, food retail, food environment, food policy

## Abstract

**Background:**

The growing dollar store sector has raised concerns about nutrition and associated health outcomes, especially for low-income communities who disproportionately rely on dollar stores. Perspectives of dollar store shoppers are largely absent.

**Objective:**

This study aimed to understand why low-income shoppers choose to purchase food from dollar stores and what store changes, policies, and programs would make it easier for them to purchase healthier items.

**Methods:**

In May–June 2023, we conducted interviews with 19 dollar store shoppers in an urban county in North Carolina. We used thematic analysis and the framework method to identify emergent patterns and themes across responses.

**Results:**

Individuals relied on dollar stores because of the affordable prices and convenient locations. In order of frequency, most participants purchased candy and snacks from dollar stores, followed by meat, fruits, and vegetables. Participants wanted more fruits, vegetables, and higher quality proteins at dollar stores and supported policies that increase access to healthier options via increased purchasing power, increased access to a mobile farmers’ market, marketing that identifies nutritionally healthy products, and improved access to other store types. Responses to removing unhealthy items from checkout areas were mixed.

**Conclusions:**

Dollar stores are affordable and convenient food retailers for people with low incomes. However, dollar stores are not meeting demand for fruits, vegetables, and proteins, items necessary for food and nutrition security. To improve food access and community health, decision makers should incorporate community perspectives into efforts aimed at improving dollar store food options.

## Introduction

In the United States, dollar stores (e.g., Dollar General, Dollar Tree) are the fastest growing food retail type by share of household expenditure [1]. In a 2013 annual report, Dollar General alone reported that, with 11,000 stores, they had, “more locations in the US than any other retailer.” [2] A decade later, Dollar General’s 2023 Fiscal Report announced that it had 19,147 locations, almost doubling its store count and expanding into international markets [3].

Although they provide food access when grocery stores are not available [3,4] and offer affordable options for low-income shoppers [5], dollar stores offer significantly fewer food options than other retail types [4] and, on average, purchases from dollar stores are less nutritionally beneficial than purchases from other food retailers [5]. Because dollar stores tend to locate in low-income neighborhoods with large Black, Asian, and Hispanic populations [[6], [7], [8]] and because low-income communities of color disproportionately rely on dollar stores for food [1], it is imperative to understand how growth in the dollar store sector influences food purchasing behaviors in these communities. This information is critical for improving diet quality and associated health outcomes as well as advancing equity by ensuring that all communities have equal access to nutritious food.

As a result of dollar store growth, inequitable placement, and limited access to fresh food options, between 2018 and 2020, >25 United States localities passed regulatory policies that prevent new dollar stores from opening or place limits on where they can open [9]. Yet largely absent from the academic literature are the perspectives of dollar store shoppers. The Center for Science in the Public Interest recently surveyed a national sample of individuals living near dollar stores and found that community members had generally positive perceptions of dollar stores [10]. However, more research is needed to inform targeted and equitable food access interventions.

Through semistructured interviews, this formative study aimed to understand why low-income dollar store shoppers in an urban area in North Carolina choose to shop and purchase food items at dollar stores and what store changes, policies, and programs would make it easier for them to purchase healthier foods there.

## Methods

### Study setting and context

This study took place in New Hanover County, North Carolina (NC), which had the highest availability of dollar stores by land area within NC at the time of this study. In 2023, there were 29 dollar stores in the county, 15% of all Supplemental Nutrition Assistance Program (SNAP)-approved food retailers, with 1 dollar store per every 17 sq km, or 1.3 dollar stores per 10,000 people. In 2020 [11], 13.1% of New Hanover County’s population were Black or African American and 6.2% were Hispanic or Latino; median household income was roughly $57,000; and 10.2% of the population had incomes below the poverty line.

This research was conducted in partnership with the non-profit organization, Feast Down East (FDE). Via a mobile farmers’ market, FDE reaches communities experiencing food insecurity by bringing nutrient-dense, affordable foods to those who would otherwise have limited food access. According to data collected by FDE, 27% of mobile market registered shoppers identify as Black or African American, 15% as White, and 8% as Hispanic or Latina/o; and 77% of mobile market sites are closer to a dollar store than a grocery store. To better understand nontraditional options for accessing food and to inform future programming, FDE expressed interest in learning more about how community members perceive and use dollar stores.

### Participants and procedures

Participants were recruited via flyers posted at the FDE Mobile Market and in shared spaces in affordable housing communities, as well as by word of mouth. Interested individuals completed an online or telephone eligibility survey.

To be eligible, participants had to shop at dollar stores at least twice per month, be the primary grocery shopper for their household, be available for a 45-min in-person interview, and be able to complete an interview in English. Semistructured interviews were conducted in-person by one author (AER) in a quiet, private space selected by each participant, such as shared spaces in affordable housing communities, community centers, and the public library. On the basis of prior research about the standard number of interviews required to reach saturation, we aimed to recruit between 16 and 24 participants [12]; however, to give as many community members the opportunity to voice their opinions as possible, participants were continuously recruited until no additional individuals expressed interest in participating.

At the beginning of the interview, the study’s purpose was explained, and participants reviewed and signed an informed consent that included consent to audio recording via digital voice recorder and Zoom Meetings software (Zoom Video Communications, Inc; Version: 5.12.9). After the interview, participants completed an online demographics survey on a portable iPad via Qualtrics and received $40 compensation. All interviews were conducted between May and June 2023. The University of North Carolina at Chapel Hill institutional review board approved procedures for this study (#23-0468; 23-3086).

### Interview guide

The interview guide was developed in consultation with FDE, community members, and the North Carolina Translational and Clinical Sciences Institute. Inspired by Glanz et al.’s [13] model of the community nutrition environment [14], the guide was comprised of the following 4 sections of questions, each of which allowed for spontaneous probing during the interview: *1*) general shopping patterns, *2*) community food environments (i.e., dollar store options), *3*) consumer food environments (i.e., product preferences within dollar stores), and *4*) perceptions of dollar store policy and program options. The policies and programs discussed were informed by a published dollar store policy scan [9] as well as conversations with community partners and policy experts. The interview guide was finalized before the first interview but allowed adaptation of the language and use of spontaneous probing.

After the interview, participants were also asked their age, race, ethnicity, gender, income, and education via online survey. Food insecurity status was assessed via the Hunger Vital Signs 2-item measure [15,16]. See Supplemental Material for the full interview guide.

### Analysis

We applied a thematic analysis [17,18] and utilized the 7-step framework method [19] which includes the following: *1*) transcription, *2*) familiarization with the interview, *3*) coding, *4*) developing a working analytical framework, *5*) applying the analytical framework, *6*) charting the data into the framework matrix, and *7*) interpreting the data.

Each recording was transcribed with the AI transcription software, Otter.Ai then checked for accuracy by AER. As the interviewer and transcriber, AER became familiar with the transcripts by reviewing them iteratively and recording analytic notes.

After data collection was complete, 2 authors (AER and EC) independently reviewed and coded the first 3 interview transcripts. To honor participant voices and experiences in this research, all codes were derived inductively from the transcripts, rather than from researcher preconceptions. After discussing and comparing initial codes, AER and EC developed a codebook (i.e., the analytic framework) that included agreed upon codes, categories of codes, code definitions, and examples of each code from the transcripts. The codebook was then updated following feedback from MGH, SDG, and a qualitative research expert. AER and EC applied the codebook to the first 5 interviews (recoding the first 3) and then reconvened to discuss any additional changes to the codebook, categorize codes under each of the 3 research questions, and discussed, and resolve discrepancies via consensus [20]. Once AER and EC were confident that there was consensus in implementing the codebook, they split the remaining 14 interviews and met to discuss questions, potential new codes, and discrepancies in codebook implementation. As part of the analysis, saturation was assessed after all interviews were complete and determined as the point, “when no additional issues or insights emerge from data and all relevant conceptual categories have been identified, explored, and exhausted” [12,21]. The codebook stabilized after the first 9 transcripts such that no additional unique codes were discovered after that point. Coding was conducted in Dedoose (Dedoose Version: 9.0.17) and after coding each interview, AER and EC wrote short narrative summaries to maintain each individual lived experience.

Once each transcript was coded, codes and illustrative quotes were charted into framework matrices. We counted the number of participants who expressed major ideas under each theme to facilitate understanding of patterns. We use the terms “few,” “some,” “more than half,” and “most” to describe themes discussed by less than one quarter, up to half, up to three quarters and more than three quarters of participants, respectively [22].

From there, the authorship team discussed the data and collectively interpreted the findings among themselves and with local experts. To aid in interpretation, AER conducted a “data party” [23] with the New Hanover County based Cape Fear Food Council. As a participatory research method, data parties create the space to share preliminary results with key stakeholders, collaboratively interpret them, and identify next steps to address the issues raised. Rather than generating new findings, the aim of this data party was to reduce researcher bias in interpreting the results. Food Council members discussed key takeaways from the interviews and their interpretation and implications based on these findings. The COREQ [24] checklist was used to ensure comprehensive reporting of all relevant information.

### Reflexivity

The research team has varying lived experiences and perspectives that both relate to and differ from the participants in this study. The lead author (AER) developed the interview guide, conducted interviews, and analyzed the data as a PhD candidate at the University of North Carolina at Chapel Hill. AER is a White American female who lives in the urban setting where this study took place and actively engages with the community but does not rely on dollar stores for food. Acknowledging the potential for bias arising from diverse lived experiences, AER attempted to approach each interview with a neutral perspective. In practice, this resulted in asking participants probing questions to fully describe their opinions and behaviors rather than assuming to know what participants meant. Moreover, the analytic process relied on inductive codes and narrative summaries to ensure that authentic participant perspectives were shared, as well as the use of the data party described above to aid in interpretation. The additional authors live in an urban area in the same state as the study area and have shopped at dollar stores occasionally but do not rely on them for food. MGH is a White American female who studies food, tobacco, and alcohol policy; her role was to provide input on study methods and manuscript drafts. SWN is an immigrant female and LST is a White American female, both of whom work on food policy issues in national and global contexts and provided feedback on interpretation and manuscript drafts. KMR is a White American professor who researches tobacco control and cancer prevention; his role was to review study methods and research findings. ELC is a Black American male public health student who added his lived and work-informed perspectives on food equity to the project. He contributed to the qualitative data analysis and manuscript editing. SDG is a White American female who studies tobacco, alcohol, and food retail environments; she advised all aspects of study design and implementation, and provided input on all manuscript drafts.

## Results

In total, we conducted 19 interviews with dollar store shoppers (Table 1). The majority of participants identified as female (79%) and their average age was 62 (range: 35–73 years). Three quarters (74%) of participants identified as Black or African American, 26% as White, and none as Hispanic. Most (82%) were living <185% of the Federal Poverty Level (FPL) for their family size. Almost half (42%) of participants reported that they purchased more than half of their food from dollar stores and two thirds (67%) were food insecure.

**TABLE 1:** Demographic characteristics (*N* = 19).

Characteristic	*n*	%
Mean age, year (range)	62 (35–73)	
Gender identity		
Female	15	79
Male	4	21
Race		
Black or African American	14	74
White	5	26
Hispanic or Latino/a	0	0
Income		
$0–$24,999	12	63
$25,000+	5	26
Do not know/prefer not to answer	2	11
Below 185% FPL^1^	14	82
Federal nutrition benefit receipt^2^		
Supplemental Nutrition Assistance Program (SNAP)	14	74
Special Supplemental Nutrition Program for Women, Infants, and Children (WIC)	0	0
Education		
Less than a HS degree	5	26
High School degree (or equivalent)	3	16
Some college (no degree)	5	26
Associate’s, bachelor’s, or postgraduate	6	32
Transportation		
Access to a personal vehicle	11	58
Travel with family or friend	7	37
Public transportation	3	16
Bike or walk	2	11
Percent of food that comes from dollar stores		
>50% (most)	8	42
<50%	11	58
Food insecurity status via Hunger Vital Signs		
Insecure	12	63
Secure	6	32
Don’t know/prefer not to answer	1	5

Abbreviations: FPL, Federal Poverty Level.

Participants could select more than one mode of transportation.

1 The 2 participants who reported “Don’t know/prefer not to answer” as their income were not included in the “Below 185% FPL” measure. There is no other missing data.

2 Major chain dollar stores accept SNAP but do not accept WIC benefits.

### Reasons for shopping at dollar stores

The most frequently reported reason for shopping at dollar stores was affordable prices, followed by convenience, the food and nonfood products offered, and needing just a few items.

#### Dollar stores are a low cost and convenient option

Most participants shared that they were struggling financially and shopped at dollar stores because of the affordability, although most items no longer cost only one dollar. One participant who purchased most of her food from dollar stores shared:

“I go in there and rack up on all of [the food], cause that ain't gonna cost me more than about $20 to come up out of there… The dollar store got it going on.”

Dollar stores increase shoppers’ limited purchasing power by enabling them to buy the items they need at a price they can afford, as illustrated in the following comment offered by another participant who also purchased most of her food from dollar stores:

“You can manage more with your funds when you go to the dollar store. Come out of the dollar store you can have $50 worth of merchandise. If you go into a regular store, you be blowing away that $50 and say, ‘Now this was $50? Got two things and this was $50?’ [At the dollar store] I got a basket with say about 45 items or something like that for $50. So you know, it's a difference. The dollar store is, to me, I think it's a blessing.”

Affordability was particularly important for the participants who noted that pandemic-related federal SNAP benefits ended in March 2023 and inflated food prices made it difficult to feed themselves and their families. When comparing dollar stores to other store types, participants most frequently shared that other stores were too expensive for their budget. Even stores traditionally thought of as affordable stretched participants’ already limited budgets, as described by the same participant who discussed the dollar store price increase:

“And Walmart is very high when it comes to grocery shopping. It’s really not a bargain there. If I have to go there then I will, but normally I don’t go there to shop for no kind of food or cleaning supplies and stuff because they’re very high.”

The sentiment that Walmart and select regional grocers known for low prices were too expensive was common throughout the interviews. Participants described themselves as bargain shoppers, comparing store prices to find the best deal, often resulting in dollar store purchases.

Convenience was the second most frequently reported reason for shopping at dollar stores, discussed by more than half of the participants. One participant spoke on “the convenience of them being in every neighborhood,” noting that dollar store omnipresence was one of the key convenience factors that drew her to dollar stores. Convenience also encompassed proximity to the participants’ home, walkability, and the ability to coordinate transportation. As one participant shared:

“For some of the elderly people [in the neighborhood] it's convenient. And it's savings for them. But I think it’s mostly the convenience because some of them can walk there, or somebody's always going to the dollar store you can get a ride. But I think for them, it's convenient.”

Participants appreciated their ability to easily access dollar stores on foot, by carpooling, or via the public bus system, particularly given that roughly half the participants in this sample did not have access to a personal vehicle and transportation was frequently cited as a general barrier in day-to-day life.

#### Dollar stores carry the products shoppers need to get by

Most participants shared that they go to dollar stores because they carry the products that they need. A participant who purchased most of her food at dollar stores described the availability of products at dollar stores:

“I like the price and the availability of things that they do have there…Anything I need, I can go right there and get it. Don't have to worry about it.”

Food products were described as comparable with products offered in other stores. One participant said, “they food is just like everybody else’s food.” Participants shared that dollar stores even carried brand name products, which were preferred but often unaffordable at other store types. Brand name was particularly important because these items are predictable and dependable. Participants expressed not wanting to waste their limited budgets on off-brand products that may taste different and thus not be eaten.

Although roughly half of participants purchased more than half of their food from dollar stores, participants shared that they often go to the dollar store for just a few items between larger grocery trips. Dollar stores were particularly helpful when participants had just a few dollars to spend or did not need the large quantities of food that are packaged together elsewhere. A participant who lives alone described the appeal of dollar stores:

“Yeah, I like shopping there because sometimes the smaller quantities are better than when you go in the major store and you have to get the big one. I like the little individuals because it's, like I said, it's just me until the grands or the great grands come over then it becomes everybody's stuff.”

#### Friends and family motivate shoppers to first try dollar stores

In addition to why they currently shop at dollar stores, participants shared their original motivations for trying them. Almost half of participants shared that they had not tried shopping at dollar stores until a friend or family member took them there. A participant who has been shopping at dollar stores for almost a decade described first shopping there because of her daughter:

“My daughter say she seen an advertisement for something in Dollar General and she wanted me to take her there. I said girl what you going in there for... She said mama I promise you, they got bargains… And ever since that day, I started going there and getting bargains. Yes I did. Yep, it was her. It was my daughter. We ended up going there all the time.”

Because of these social connections, participants found that the items they typically purchased were available at dollar stores for more affordable prices. Continuing to visit these stores with friends and family seems to have fostered social momentum, establishing a community norm around purchasing food from dollar stores and facilitating transportation to them.

### Purchases at dollar stores

#### Snacks, meal essentials, and household supplies

Most participants shared that they purchased snacks and candy, meat, and fruits and vegetables from dollar stores. More than half described purchasing grains, nonfood items, and dairy products, and some shared that they purchased beverages, frozen meals, cooking essentials, and plant-based proteins such as beans. The descriptions of food purchased at dollar stores fell into 2 categories: snacks/candy and meal essentials. A participant who took her grandchildren to dollar stores described their snack food purchases:

“They don’t buy nothing that’s nutritious really. But they gonna wish they had. They love them sweets. They really do. And they love them chips.”

All participants shared that they purchased more than just snacks and candy from dollar stores. A 66-y-old participant who purchased more than half of her food from dollar stores and lived alone shared, “I like dollar stores. I mean, if I had to get a meal with that, I could make a meal out of there.” Even participants with larger families shared that they could rely on dollar stores for simple meals to help their family. A participant with a household of 4 described his purchases as:

“…the canned goods, the eggs, bread, you know, necessary stuff, something for the [kids] to get going and fix a sandwich with real quick.”

Meat products were discussed by almost every participant. In order of frequency, these products included canned meats (e.g., Vienna sausages), lunch meats, refrigerated and frozen sausage, canned seafood (e.g., tuna and salmon), frozen meat (e.g., chicken, seafood, beef), and hot dogs. A participant who purchased more than half of her food from dollar stores explained that the few meat products that were less processed, such as frozen cuts of chicken, were often sold out or difficult to find:

“They don't have too many types of meats and they go so quick, baby. The meats go quick. But they have a lot of like, you know stuff you can put in the oven…they have hamburgers, they have chicken, they have chicken breast, chicken legs.”

Participants frequently reported purchasing canned fruits and vegetables including corn, green beans, tomatoes, and peaches, pears, and pineapple. Fewer participants purchased frozen fruits and vegetables, noting that the frozen options were high quality and preferable to canned options but sold out quickly at the few dollar stores that stocked them. A participant experiencing food insecurity said that she would buy large quantities of frozen vegetables from dollar stores when they were stocked:

“I get spinach…whenever I can find it I go and get about four or five bags. Because they sell out of it fast. Yeah. And then if there's any other vegetable I'll just pick it up.”

The additional foods participants reported purchasing at dollar stores were described as pantry staples that were cheaper at dollar stores than at other stores, even when there were nationwide shortages. These included grains such as breakfast cereals and grits; dairy products such as milk and eggs; beverages such as soda, juice, and water; frozen, ready-to-heat meals such as frozen pizza, TV dinners, and sides; cooking essentials necessary to complete a home cooked meal such as seasonings and sauces; and plant-based proteins such as canned and dried beans. Participants were cautious when purchasing frozen and refrigerated items and often checked expiration dates.

In addition to food, more than half of the participants purchased nonfood items. These were most often household supplies such as paper products, cleaning supplies, food preparation gadgets, and storage containers; personal toiletries; home décor such as silk flowers and vases; and greeting cards.

#### Shoppers often make unplanned purchases

Participants cited being able to quickly run in for a few items as a reason for shopping at dollar stores. However, these participants rarely left the dollar store with just the items they intended to purchase. All participants shared that they, along with their friends and family, made unplanned purchases when shopping at dollar stores. A participant who purchased more than half of her food from dollar stores said:

“When I go into the [dollar] store, even what's not on the list, I end up getting. And I be telling the [dollar store employee], I say, I came in here just for this, and I end up getting all of this. I said, it never fails. Why? Why do I do this?”

Unplanned items most often included candy and snacks, as well as household supplies, entertainment products, and grains. Shoppers at dollar stores had trouble avoiding unplanned items they enjoyed or needed, considering the low prices that empowered them to indulge without exceeding their planned budget. Compared with other stores, participants seemed to be more inclined to make unplanned purchases at dollar stores, as a participant who purchased less than half of her food at dollar stores shared:

“Oh, yeah. Especially at the dollar store. Because you're always seeing things that you know you want…and when they're that cheap, you kind of can justify it.”

#### Items for family and friends

More than half of participants talked about shopping at dollar stores for people in their lives. Because of the low cost at dollar stores, participants could purchase items for friends, family, and even strangers. For family members, this looked similar to being able to accommodate large households, grown children when they came home to visit and extended family members, such as grandchildren.

Dollar store shoppers additionally supported friends and social networks with their dollar store purchases. This extended to elderly neighbors who could not easily shop for themselves, social circles of people who could not easily access affordable food, and religious congregations with limited resources. Participants expressed a selfless desire to help their community and gratitude for the dollar stores that made it possible. A participant who managed the community space in her housing authority community described purchasing items for everyone to share:

“We have a soda machine out here [gestured to a vending machine] and I tell the [residents], you don't go [to the vending machine] because it's $1.75 for a soda and $1.75 for some chips. I say, you can go to the store and buy a whole pack or the box with the different chips in it, and then you can get four six packs of sodas for like $12 or $10… So I buy things and bring them here. I catch the sales and put it here so we have sodas.”

These efforts to support the community extended beyond friends and family. A participant currently experiencing housing instability and food insecurity described dollar store shoppers and their desire to help others get by:

“I went shopping and ran into this woman… she pulled me aside and she was like, hey I see that you're always kind of in here… let me help you out… It just seems like people who go into the dollar stores are friendlier. Kind of willing to always help somebody. I mean, if somebody's like, short, it's $1. So you know, it's like not a huge deal versus another store, it might be like $20 over or something. So I think that's a huge thing… I've seen it, I've actually witnessed it.”

These experiences collectively depict individuals engaging in acts of community kindness and social support through their shopping habits, enabled by the affordable prices at dollar stores. Participants express a willingness to share purchases with family members, pick up items for neighbors, and contribute snacks and drinks to community centers, showcasing a sense of generosity and community support.

### Ways to improve dollar stores

Most participants made suggestions for dollar store improvements. The most frequent suggestions were to increase fruit and vegetable options, improve the quality of meat products offered, expand overall variety, keep items stocked, and generally introduce healthier food options.

#### More fruits, vegetables, and higher quality proteins

The most frequent suggestion was to increase the amount of fruits and vegetables offered. Participants were enthusiastic about the prospect of accessing fresh fruits and vegetables at dollar stores, as detailed by the participant who took her grandchildren to dollar stores:

“They got the major canned fruits, they do, but I just wish they had, you know, fresh fruit in there, because I would be going in 24/7 for the grapes and stuff. I really would. They haven’t got there yet.”

A few participants additionally discussed fresh fruit and vegetable sourcing, suggesting that dollar stores could “have a partnership with the local growers.” However eager, participants understood the barriers to stocking fresh produce. Unprompted, they discussed the downsides of introducing fresh produce to dollar stores, including limited shelf space and food waste concerns. A participant who purchased less than half of her food from dollar stores shared:

“I think it would be great [if dollar stores sold fresh produce] but it's the size of the stores. Because in my experience…it's just, where are you going to put it? But I mean, I think it'd be great if I could go, ‘okay, they have this or that’.”

This realism translated into an interest in increasing the quantity of frozen fruits and vegetables, rather than focusing solely on fresh options. A participant said:

“I’d like to see more variety in the frozen, like not just corn, green beans, have different types... I know there's not a lot of people who eat brussels sprouts and things like that, but I do. And I'd love to see them carry things like that. You know, just a variety of different vegetables.”

In addition to suggestions for fruits and vegetables, participants expressed a desire for higher quality meat in dollar stores. Almost half of the participants either described the meat as “not real” or “not fresh,” or shared stories of seeing out-of-date items on shelves, getting sick after eating them, or avoiding certain items because they had been recalled in the past. A participant who purchased more than half of her food from dollar stores shared:

“As far as buying meats, they don’t have a good selection...those hot dogs ain’t real. I mean they're real, but you know what I'm saying. I want the beef hot dogs, and I don't see that in there. I see the chicken/pork product.”

A small number of participants used broad terms such as “healthy food” when suggesting changes that dollar stores could implement. A participant living below 185% of the FPL and experiencing food insecurity described how stocking healthier items at dollar stores would encourage the community to purchase healthier items:

“Just to have more healthy foods. They have a lot of prepackaged foods, because of us. A lot of people, especially in this area buy... But I think if they would carry fresher, people would buy fresher. Yeah. If you don't see it, you're not gonna buy it.”

#### In-store structure and logistics

A few participants specifically requested that dollar stores keep their shelves stocked. Almost half of the participants indicated that dollar stores were frequently disorganized with poorly stocked shelves, making it difficult to find the food they needed. A participant who purchased more than half of her food from dollar stores described the last time she shopped at a dollar store as frustrating because of the disorganization:

“I didn’t get any food and half the stuff was in the middle of the floor. It was cluttered. The stuff that was supposed to be on the shelf was not on there. [I] asked where the stuff was at and [the employee] said, ‘I really don’t know cuz there’s so much stuff around that needs to be put on the shelf’. So I had to rump around a lot of boxes and all that stuff like that.”

After getting their items, some participants discussed waiting in long lines to check out. Given that convenience was a common reason for shopping at dollar stores, participants expressed frustration with occasional lines and long wait times. As the participant who managed the community space in her housing authority community described, this was due to the large number of people who shop at dollar stores coupled with only one checkout employee at a single register:

“Only thing sometimes I dislike about shopping at dollar stores is for the lines to get so long. Because they have so many good savings there and everybody wants to buy and everybody wants to check out and they may have two registers but only one working. And that's the problem there. Or you got somebody there who doesn't know what they doing and it's so slow and you can sit there and maybe take a nap, by the time you wake up, they still doing the same thing.”

In-store logistics issues were blamed on limited staffing by multiple participants. When asked directly about the changes that dollar stores could implement, a participant who purchased more than half of her food from dollar stores asked, “Could you pay people more wages and have more workers?”

Although most participants wanted to see improvements made at dollar stores, it should also be noted that all participants shared that they greatly appreciated dollar stores and felt safe shopping at them. When asked if there were ways that dollar stores could change to make it easier to eat a healthy diet, one participant shared her admiration and dependence when she said, “no, just don’t take them away.”

#### Policies and programs that increase fresh food access

Finally, participants indicated their support for various policy and program options that aim to address dollar stores and improve food access. Because this question was asked at the end of the interview, not all participants had time to discuss each option. In addition to the following text, the number of participants who provided feedback on each policy or program as well as approval and illustrative quotes for each can be found in Table 2. All participants who were asked about providing discounts on fruits and vegetables purchased with SNAP benefits (i.e., dollar-for-dollar SNAP matching Fresh Bucks in NC) or a proposed program to bring FDE Mobile Famers’ Market to dollar store locations endorsed these options emphatically.

**TABLE 2:** Policy/program options and their approval among participants.

Policies and programs	No. of participants who discussed policy/program	Participant approval	Additional illustrative quotes
1. Provide discounts on fruits and vegetables purchased with SNAP benefits at dollar stores (i.e., Fresh Bucks)	16	100% approved 0% mixed 0% disapproved	“Yes. Yes I agree. Because that will be a bonus for everyone that have SNAP and EBT. It would be a bonus. Yes.”
2. Bring the Feast Down East Mobile Farmers' Market to dollar store locations	8	100% approved 0% mixed 0% disapproved	“[With] the truck! Oh, yeah, that'd be a great idea. Yeah, yeah, that's smart.”
3. Require dollar stores to advertise healthier food options in the store	12	92% approved 8% mixed 0% disapproved	“Yeah. I think if people knew they had [healthier food], more people would go… If they knew they had them at the Dollar Tree, they could just run up there and grab what they needed.”
4. Improve transportation to other food retailers and/or add bus stops and quick routes to traditional grocery stores	15	80% approved 7% mixed 13% disapproved	“They do need that for elder people. So a lot of people don't have a car. And then people charging you too much to take you somewhere and then sometimes people don't got time.”
5. Require new dollar stores to sell healthy food options in order to open (i.e., conditional use ordinance)	16	69% approved 19% mixed 13% disapproved	“Yes. I'm not one to talk about being a good, healthy eater. But I do try. It's difficult when [unhealthy food] all that you see you can buy and [unhealthy food] is cheaper.”
6. Require new dollar stores to sell fresh fruits and vegetables in order to open (i.e., conditional use ordinance)	18	67% approved 17% mixed 17% disapproved	“Yeah, that's a good idea…. I know cabbage would sell. Cabbage and some broccoli. Right? Yeah, and some fresh green beans. Yeah, stuff like that. Fresh corn. People would buy that too.”
7. Remove less healthy food products from the checkout counter/line (i.e., healthy checkout)	17	41% approved 24% mixed 35% disapproved	“No, no, because everybody need a piece of candy every now and then... Don't take them away because it's what you want. You don't have to buy it, pass by... That's why I tell people, don't cramp my lifestyle because of your lifestyle.”

Abbreviation: SNAP, Supplemental Nutrition Assistance Program. EBT, Electronic Benefits Transfer.

Most of the people who discussed requiring dollar stores to advertise and promote their healthier options expressed support, although one participant was unsure if the option would make a big difference. After learning about this option, a participant living <185% the FPL and experiencing food insecurity described how it could increase informed decision making:

“And that's still giving the option if they want to get the healthy food or get the other regular food. You know what I'm saying? So give them the options and let them know. It's their choice.”

Most of the participants approved of improvements that could be made to public transportation that would make it easier to get to other store types (i.e., grocery stores), but one participant was unsure and two disapproved of it. Of note, these three participants did not use public transportation frequently, if ever.

More than half of the participants who learned about conditional use zoning ordinances requiring new dollar stores to stock either fresh fruits and vegetables or, more generally, healthy food, endorsed these policy options. Three participants were unsure of one or the other and three and two participants, respectively, did not support the policy options altogether. Opposition centered around not wanting to intervene in the free market. A participant who previously worked at a dollar store chain described a sense of uneasiness when hearing about policy requirements for stores:

“If they feel they can find a way… to be able to market it. I still think it should be an option. But if they could find a way to be able to do it and test it out and run it and see if there's a market for that. But I hate to hear the 'well you're required to carry'…Required in store, that gets me in that funny slippery slope feeling.”

Finally, opinions were divided regarding the healthy checkout policy option. Although more participants approved than disapproved, one participant shared: “That's where the little kids get their little excitement from. My little kids love them aisles, so, let the little kids have some fun,” indicating that being able to purchase items for their children at checkout was an enjoyable experience. All the disapproval for the healthy checkout policy option centered around not wanting enjoyable items, such as candy bars, taken away from a highly visible location in the store. As noted in Table 2, one participant expressed her disapproval by sharing, “no because everybody need a piece of candy every now and then... Don't take them away because it's what you want.”

## Discussion

In this qualitative study, we shed light on the various reasons why low-income shoppers in an urban setting use dollar stores, what they purchase from these stores, and how they would like to see the stores improved. Our findings emphasize that dollar stores are cost-effective and convenient options for households to purchase their snack foods, meal essentials, household supplies, and items for other people. This study also found that dollar store shoppers purchase a variety of food items from dollar stores, depending on them for meal essentials while impulsively buying snacks and other items for entertainment. Finally, our results indicate that shoppers support programs and policies that increase their purchasing power and fruit and vegetable options.

Our findings are consistent with research from the broader food environment literature, including a report from the Center for Science in the Public Interest [10] as well as previous research citing convenience and proximity to home, work, or other spaces and low prices [[25], [26], [27], [28]] as the reason for shopping at non-traditional food retailers such as dollar stores, convenience stores, and gas stations.

Within NC, dollar stores may be convenient food retailers for individuals living in affordable housing communities in part due to tax credits that incentivize developers to build low-income housing near dollar stores. Since 2012, the North Carolina Housing Finance Agency has provided housing credits and tax-exempt bonds to low-income housing developments built within 2 miles of a grocery store, pharmacy, and general shopping (which includes dollar stores) as detailed in annual Qualified Allocation Plans [29]. Thus, this program may result in low-income housing communities that are close to dollar stores, with limited fresh food options. Both statewide and nationwide studies are needed to determine how these tax credits impact dollar store proximity and food access for low-income housing communities.

Similar to existing research across varying food environments [[30], [31], [32]], our findings underscore the significant role of social connectedness in shaping shopping behaviors and experiences of individuals with low incomes. Participants consistently highlighted the influence of friends and family as their motivation for shopping at dollar stores and the dollar store’s affordable prices as the reason why they could purchase items for others. It is worth noting the social connectedness that emerged from our interviews may be a result of participant age. The average age of participants in our study was 62 year old, with just over half of the participants reporting ages >65 year old. Older adults report that in-person grocery shopping is an activity they engage in specifically to socialize with others [33,34], which may have been why various social behaviors were reported throughout the interviews. Regardless, as dollar stores continue to establish their importance in the social fabric of low-income communities, loyal customers might not realize how these stores could be fostering unhealthy purchasing habits, especially if they feel that healthier alternatives are unattainable.

Consistent with the Center for Science in the Public Interest report and other research [10,25], participants reported purchasing a variety of items from dollar stores including snacks and candy, as well as meal essentials such as proteins (most often processed meat), canned or frozen fruits and vegetables, grains, and dairy products. Many of these items often contain high levels of nutrients of concern such as sodium, saturated fat, and sugar. This again is consistent with existing research finding that purchases from dollar stores do not align with federal nutritional recommendations [5]. The consumer food environment prompting impulse purchases may partially be to blame. Similar to what participants reported, unplanned impulse purchases are typically comprised of hedonic foods that often contain excessive nutrients of concern [5,[35], [36], [37]]. Purchasing impulse items may be a more common behavior at dollar stores than other stores because, as participants shared and prior research supports, affordable pricing and price discounting encourages the behavior [38]. These findings indicate that dollar stores play a role in the interrelated concepts of food security, “access to enough food for an active, healthy life including the ready availability of *nutritionally adequate and safe foods*,” and nutrition security, “the consistent and equitable access to healthy, safe, affordable *foods essential for optimal health and well-being*,” [39,40]. Although we found that dollar stores offer convenient and affordable food options, two important components of food security, they do not appear to support nutrition security to the extent that they could. As participants in this study shared, dollar stores do not stock enough foods essential for optimal health such as fresh or frozen fruits and vegetables or high-quality proteins. Future research should specifically evaluate if and how dollar stores may impact food and nutrition security differently. This is of particular importance in light of 1000 Dollar Tree/Family Dollar closures [41] that may inequitably impact food and nutrition security.

Participants expressed a desire for healthier items, specifically fresh and frozen fruits and vegetables and higher quality meat, consistent with other research [10,26], suggesting an opportunity for intervention. In early 2024, Dollar General reported that they offered fresh produce in 5000 stores [42]. However, the quality and quantity actually available in stores has yet to be verified by an external source. To meet shopper demands while improving the nutritional benefits of the food offered, dollar stores should stock more fresh and frozen fruits and vegetables and higher quality proteins.

In addition to specific food items, dollar store shoppers also wanted to see improvements made to the store more generally, such as better stocked shelves and more efficient checkout lines. Similar store frustrations were documented in an ethnography from the perspective of a dollar store employee who cited that operating the store, including stocking shelves and checking customers out, was nearly impossible due to purposeful and persistent understaffing [43]. Dollar stores also typically only pay the minimum wage and expect managers to work unpaid overtime [44]. Dollar store chains may want to explore ways to increase or better incentivize staff. This approach could both make it easier for shoppers to get the food they need and support local communities with favorable employment opportunities. As decision makers and researchers design and study programs and policies that aim to increase food access by engaging dollar stores, the costs of those policies, and whether those costs would be passed on to consumers if implemented, needs to be considered, to ensure that the price point at dollar stores remains affordable for households with limited resources.

We found a great deal of congruence in approval for specific policy and program options between dollar store shoppers in this study and participants in the Center for Science in the Public Interest report [10]. The highest level of support was given to expanding the SNAP dollar-for-dollar matching program (i.e., Fresh Bucks) that would double SNAP purchasing power when used to buy fruits and vegetables. Financial incentives for low-income shoppers to purchase fruits and vegetables are popular intervention mechanisms across the United States, typically have high user satisfaction, and are proven to increase fruit and vegetable purchasing [[45], [46], [47]]. However, such programs are often restricted to use at farmers’ markets and food co-ops, if they are available at all [48]. To increase access to these programs, localities could consider including additional retail types such as traditional grocery stores, as has been found feasible and acceptable in select United States cities [49,50], and possibly dollar stores. Furthermore, localities could advocate for statewide SNAP dollar-for-dollar matching programs, providing equitable access for all state residents. This type of program may also offset potential costs passed on to the consumer from the improvements made to dollar stores.

Policies with the most support from this sample would directly increase access to healthier options via increased purchasing power, increased access to fruit and vegetables through a mobile farmers’ market, promotions and marketing that educates shoppers by identifying nutritionally healthy products, and improved access to other store types. Policies with the least support required dollar stores to sell healthy options or fresh fruits and vegetables and to remove less healthy options from the checkout. Some participants expressed mixed emotions or directly disapproved of these policies, perceiving them as instructing businesses how to operate. Often though, requirements placed on industries may be needed to enact change. When left to self-regulation, industry actors can opt-out, permit low standards, and reject transparency [51]. Because of this incongruence, policymakers should take community member feedback into account, either prioritizing more acceptable strategies or working with community members to arrive at acceptable, yet effective, strategies. How policies are described can also impact the degree of public support they receive [52]. For example, implementing a healthy checkout policy had the lowest approval among dollar store shoppers in this sample, which is surprising given that existing healthy checkout policies have been based in grassroots, community efforts [53]. Our results may be a reflection of the policy framing that described the policy as removing options from the checkout rather than improving them.

Finally, this research was motivated by concerns over the nutritional content of food available at dollar stores. However, the impact of food extends beyond nutritional content as it is steeped in culture, community, and positive emotions such as satisfaction and enjoyment. Not all individuals view nutrition as a priority above and beyond other responsibilities in their day-to-day lives, including allowing their children to choose foods high in sugar as a treat. When conceptualizing food access interventions, researchers, decision makers, and other individuals in positions of power must be mindful of how nutrition and health are defined and prioritized by different communities.

The strengths of this study include engagement with the community through partnering with FDE and data interpretation with the Cape Fear Food Council. However, the small geographic region in which this study was conducted may limit the generalizability of our findings. Individuals living with different food environments, particularly rural areas or areas with different dollar store availability, may use dollar stores for different reasons and may support different policy and program options. Living in a rural area is associated with higher food spending at dollar stores [1,54]; thus, this work could be extended via interviews with low-income dollar store shoppers who live in rural areas. Furthermore, the county this study took place in has a small Hispanic population compared with the United States and there were no Hispanic participants in this study, so the generalizability of study findings to Hispanic shoppers is unknown. Future work should assess how dollar store use and policy and program approval varies for this population. Finally, this study assessed shopper perspectives, not what shoppers actually purchased or what was actually available in dollar stores. Future research should evaluate the foods available in dollar stores to better understand how they influence the food environment.

In conclusion, dollar store shoppers with limited resources in this study appreciated the affordability and convenience that dollar stores offer. They purchased a variety of food ranging from snacks to meal essentials and desired an increase in fruits and vegetables as well as higher quality protein options in dollar stores. Findings can inform local policy and program efforts. Decision makers should incorporate community opinion when planning policy and program interventions to improve the food options offered at dollar stores.

## Author contributions

The authors’ responsibilities were as follows – AER: conceptualization; AER, ELC: data curation; AER, ELC: formal analysis; AER: investigation; AER, MGH, SWN, LST, KMR, SDG: methodology; AER: project administration; AER: resources; SDG: supervision; AER, ELC: validation; AER: visualization; AER: writing—original draft; AER, MGH, SWN, LST, KMR, ELC, SDG: writing—review and editing; and all authors: read and approved the final version of this manuscript.

## Conflict of interest

The authors report no conflicts of interest.

## Data Availability

Data described in this manuscript will not be made available because of the small sample size and risk of breaching confidentiality. The data collected for this study are not publicly available.

## Appendix A Supplementary data

Multimeia component 1 Multimeia component 1
