# Supplementary material for: “The Dollar Store Got It Going On”: Understanding Food Shopping Patterns and Policy Preferences among Dollar Store Shoppers with Low Incomes
Source: Curr Dev Nutr. 2024 Sep 13;8(10):104457. doi: 10.1016/j.cdnut.2024.104457 (PMC11780371; doi:10.1016/j.cdnut.2024.104457)
Supplement: Multimeia component 1 [file mmc1.docx]

**Interview guide**

The interview guide below describes, in detail, the themes and specific questions that participants will be asked.

We will be conducting semi-structured interviews. Questions asked may differ based on the conversation but will be related to the topics included in this guide. **The interviewer may adapt the language below and use spontaneous probing during the interview.**

**Introduction  - (Avg. time during test interviews: 3 min)**

Thank you for joining me today. My name is Allie and I’m a PhD student at UNC Chapel Hill. I invited you here today because I’m interested in learning about your thoughts and opinions on grocery shopping at dollar stores like Dollar General, Dollar Tree, and Family Dollar.

Our discussion should last about 30-45 minutes. There are no right or wrong answers to any of the questions I’m going to ask you, you’re the expert here and I’m hoping to learn from you.

Because I’m here to listen, my goal is to be quiet when you’re talking but I am also happy to try and clarify any questions you have along the way.

Before we start, there is some information that I’d like to cover:

1.**Confidentiality**: Everything that you say here will be kept strictly confidential. Nothing you say will ever be associated with you by name.

2. **Voluntary Participation**: Participating in this interview is entirely voluntary. You don’t have to answer any questions that you don’t want to answer, and you can stop the interview at any time with no consequences. The consent form you signed provides more detailed information about confidentiality and voluntary participation. Before we get any further, do you have any questions about the consent form or the study in general?

3. **Audio-Recording**: With your permission, this session will be audio recorded so that I can write an accurate report of what you say without including your personal details. Do you agree to having our discussion recorded?

**[If participant says no, they do not consent to recording]**

[NO] – Ok, that is totally fine. I know there are different comfort levels with recording, and I respect your choice. We won’t be able to proceed with this interview, but the most important thing is that you’re comfortable, so I’m glad you shared your preferences. Do you have any questions for me before we finish? Have a good day, [name], and thank you for your willingness to share your time with me.

**[If participant says yes, they do consent to recording]**

**Thanks**: Thank you for arranging your schedule today to be here for this session. I really appreciate you sharing your time and opinions about how, where, and why you grocery shop at dollar stores.

Do you have any questions before we get started?

**[If participant says no, then move on to the recording]**

Great, now we can get into the interview and I will begin recording.

**[Start audio recording: zoom – Top left bar – Meeting – record to cloud]**

First, I’d like to ask you a few general questions.

## **Interview Questions.**

**Section 1: General - (Avg. time during test interviews: 3 min)**

[Read: First I’m going to ask you some questions about your normal grocery shopping habits at all stores, not just dollar stores.]

1. To begin, can you tell me about how you decide it’s time to shop for food?
   1. *How often do you shop for food?*
   2. *Who do you shop for when you’re shopping for food?*
2. Now imagine you’ve decided it’s time to shop for food. What, if anything, do you do to prepare for your grocery shopping trip before leaving to go to the store?
   1. *Do you take inventory of the pantry / fridge?*
   2. *Do you typically make a shopping list?*
   3. *Do you typically check your bank account, cash, or SNAP/WIC/EBT funds?*
   4. *Do you search for store promotions online or in advertisements?*
   5. *Do you coordinate transportation?*

**Section 2: Community food environment (Store type options) - (Avg. time during test interviews: 5 min)**

[Thank you for sharing. Now I’m going to ask you some questions about where you usually buy your groceries]

1. When you grocery shop, how do you decide which types of store to visit?
   1. Probes:
      1. *What makes you decide to go to a dollar store instead of a different type of store?*
      2. *When would you decide to go to a different type of store instead of a dollar store?*
   2. Additional probe: *How does ________ impact your decision to shop at different stores?*
      1. *Price*
      2. *Convenience*
      3. *Location*
      4. *Transportation*
      5. *Shopping for only a single / few items*
      6. *Safety*

1. New Hanover County has almost 30 dollar stores. If you go to a dollar store, how do you decide which dollar store to go to for groceries?
   1. *How does ________ impact your decision to shop at different dollar stores?*
      1. *Location (proximity to home, work, school, etc)*
      2. *Transportation*
      3. *Chain / brand preference*
      4. *Safety*

**Section 3: Consumer food environment – Dollar Stores (Within store options) - (Avg. time during test interviews: 12 min)**

[Thank you for sharing. Now I’m going to ask you some questions specifically about grocery shopping at dollar stores]

1. Think about the last time you went to a dollar store to buy food. Can you describe your experience shopping for food there, from the time that you entered the store to when you checked out?
   1. *What was the reason you went to the store?*
   2. *What did you see when you first walked in?*
   3. *Which aisles did you go to while you were shopping there?*
      1. *Frozen food?*
      2. *Canned goods?*
      3. *Non-food aisles like toiletries, paper products, school or party supplies?*
   4. *When did you decide you were ready to check out?*
   5. *Can you describe the items you saw at the checkout? Did you purchase any of these items?*
2. Now I want you to think about all of the times you went to dollar stores to purchase food over the past month.
   1. *Did you typically bring a grocery list with you when shopping at dollar stores?*
   2. *How often did you purchase items that you hadn’t planned on purchasing? What were those items?*
      1. *Were there ever any food or beverage products advertised, promoted, or prominently placed in the store? Do you ever purchase these items?*
      2. *Can you describe the items you typically saw at the checkout? Did you ever purchase those items?*
   3. *How did you typically pay for your items?(cash, check, SNAP, etc)*
   4. *How much time did you typically spend in the dollar store?*
   5. *Did you typically feel safe in the dollar stores in the area?*
3. Think about your shopping bag after leaving the store. Tell me about the foods you buy most often from the dollar store.
   1. *What about the foods you choose not to buy from the dollar store?*
      1. *Fruits and vegetables*
      2. *Dairy*
      3. *Bread*
      4. *Meat products*
      5. *Pantry food items (e.g., pasta, canned food, spices, sauces, cereals)*
      6. *Ready-to-eat items (e.g., microwave meals, pre-made sandwiches/wraps, packaged salads)*
      7. *Frozen food*
      8. *Salty snacks*
      9. *Sweet snacks*
      10. *Drinks*
4. When you are shopping at dollar stores, how do you decide what food to purchase?
   1. *Do you ever buy food from dollar stores specifically because they are advertised, promoted, or on sale?*
   2. *Do you ever buy food at dollar stores based on the _______?*
      1. *Quality*
      2. *Brand*
      3. *Size*
      4. *Price*
      5. *Labels and other elements on the packaging*
      6. *Nutrition information*
   3. *Do you ever buy food at dollar stores because _______?*
      1. *They are familiar*
      2. *You enjoy the taste*
      3. *You have money to spend / cash on hand*
      4. *You are buying items for someone else?*
5. How do the types of food you buy from dollar stores compare to the types of food you buy from other stores?
   1. *Do you ever decide to buy food at other stores instead of dollar stores because of the _______?*
      1. *Quality*
      2. *Brand*
      3. *Size*
      4. *Price*
      5. *Labels and other elements on the packaging*
      6. *Nutrition information*
6. What do you think of the fruits and vegetables (fresh, frozen, and canned) available at Dollar Stores?
   1. *Do you purchase any of them? Why or why not?*
   2. *Would you like to see anything different?*
7. Think back to when you started regularly shopping at dollar stores. What motivated you to start shopping there?
   1. *When was that?*

**Section 4: Dollar store perceptions - (Avg. time during test interviews: 10 min)**

[Great, thank you for telling me that. Next we’re going to talk about your thoughts on dollar stores.]

1. Thinking about the people in your community, how would you describe a typical dollar store shopper?
   1. *What do you think is the typical _______ of a dollar store shopper?*
      1. *Age*
      2. *Income*
      3. *Gender*
      4. *Purchases*
      5. *Trip purpose*
      6. *Transportation method*
2. What do you like about shopping at dollar stores for food?
   1. *Think about the past month or so, can you share an example of a positive experience while shopping at a dollar store?*
      1. *Can you think of anything else you like?*
3. Are there things that you dislike about shopping at dollar stores for food?
   1. *Again, think about the past month or so, can you share an example of a negative experience while shopping at a dollar store?*
      1. *Can you think of anything else you dislike?*
4. If you could make suggestions to your local dollar store, what would you say about the kind of food they carry?
   1. *What do you wish they would carry that they don’t already?*
5. Have you ever suggested to a dollar store manager or staff member that you’d like them to carry a certain type of food? If so, what was the food?
   1. *What was your reason for asking them to stock it?*
   2. *What was their reaction?*
6. What changes do you think could be made to dollar stores that would make buying healthier food easier?
   1. *What else do you think dollar stores could do to make it easier to buy healthier foods?*
   2. *Imagine you’ve just been given full control of a dollar store in town. What’s the first thing you would change?*
7. I’m going to tell you about policies and programs that other places in the United States are using to improve dollar stores and food access. After each one, please tell me if you think they would make purchasing healthier food easier for you.
   1. *Policy options:*
      1. *Require new dollar stores to sell fresh fruits and vegetables in order to open*
      2. *Require new dollar stores to sell healthy food options in order to open*
      3. *Remove less healthy food products from the checkout counter/line*
      4. *Require dollar stores to advertise healthier food options in the store*
      5. *Improve transportation to other food retailers / add bus stops and quick routes to full-service grocery stores*
      6. *Provide discounts on fruits and vegetables purchased with SNAP/EBT benefits at dollar stores*
   2. *Program options:*
      1. *Bring FDE’s Mobile Market to dollar store locations on certain days of the week*
      2. *~~Give out food vouchers / food prescriptions that could be used at dollar stores~~*
      3. *~~Work with dollar stores to stock healthy, frozen, prepared meal options (such as Good Bowls out of UNC)~~*

**Section 5: Food Rx - (Avg. time during test interviews: 5 min)**

**[Skip if interview is running longer than 45 minutes]**

[That’s really helpful to know, thank you for sharing. Now, in this last section, I’m going to ask a few general questions unrelated to dollar stores.]

1. How would you describe the word “health” to someone who hasn’t heard of it before?
   1. What does healthy mean to you?
2. Are you interested in improving your/your household’s health? If so, what are some ways that you would like to improve you/your household’s health in the next year?
3. Are there certain types of food that you often avoid buying?
   1. *Why do you avoid buying those foods?*
4. Are there other foods you would like to buy more often?
   1. *What barriers do you have that keep you from buying them more often?*

**Thank you**

**[Turn recorders off]**

Okay, the recorders are off. Thank you for taking the time to meet with me today. We’re almost done. If you don’t mind answering a few final questions on this iPad, I’ll get you Mobile Market vouchers ready.

**[Give participant iPad with Qualtrics survey and add dates to FDE Mobile Market vouchers]**
